# The Crosstalk between Nephropathy and Coagulation Disorder: Pathogenesis, Treatment, and Dilemmas

**DOI:** 10.1681/ASN.0000000000000199

**Published:** 2023-07-24

**Authors:** Zhiwei Qiu, Xiaocong Pang, Qian Xiang, Yimin Cui

**Affiliations:** 1 Department of Pharmacy, Peking University First Hospital, Beijing, China; 2 Institute of Clinical Pharmacology, Peking University First Hospital, Beijing, China

**Keywords:** AKI, CKD, dialysis, ESKD, IgA nephropathy, kidney transplantation, nephropathy, nephrotic syndrome, platelets, thrombosis

## Abstract

The interaction between the kidney and the coagulation system greatly affects each other because of the abundant vessel distribution and blood perfusion in the kidney. Clinically, the risks of complicated thrombosis and bleeding have become important concerns in the treatment of nephropathies, especially nephrotic syndrome, CKD, ESKD, and patients with nephropathy undergoing RRTs. Adverse effects of anticoagulant or procoagulant therapies in patients with nephropathy, especially anticoagulation-related nephropathy, heparin-induced thrombocytopenia, and bleeding, seriously worsen the prognosis of patients, which have become challenges for clinicians. Over the decades, the interaction between the kidney and the coagulation system has been widely studied. However, the effects of the kidney on the coagulation system have not been systematically investigated. Although some coagulation-related proteins and signaling pathways have been shown to improve coagulation abnormalities while avoiding additional kidney damage in certain kidney diseases, their potential as anticoagulation targets in nephropathy requires further investigation. Here, we review the progression of research on the crosstalk between the coagulation system and kidney diseases and systematically analyze the significance and shortcomings of previous studies to provide new sight into future research. In addition, we highlight the status of clinical treatment for coagulation disorder and nephropathy caused by each other, indicating guidance for the formulation of therapeutic strategies or drug development.

## Introduction

**Figure 1:**
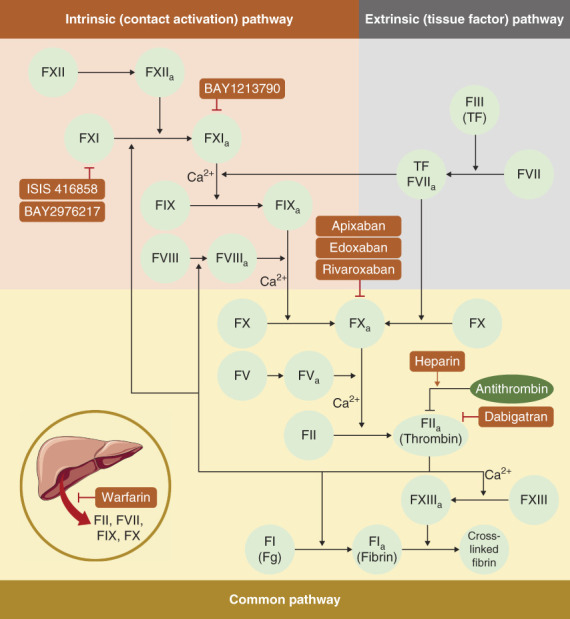
**Coagulation cascade and the targets of anticoagulants.** The coagulation process consists of a cascade of coagulation factors. Coagulation is initiated by both intrinsic and extrinsic pathways. In the intrinsic pathway, negatively charged surfaces, such as phospholipids and polyphosphates from activated platelets, activate FXII, triggering cascade reactions that ultimately activate FX. In the extrinsic pathway, tissue injury or inflammatory factors induce the expression of TFs that form complexes with activated FVII, thereby activating FX. Activated FX promotes the production of thrombin, which catalyzes fibrinogen (Fg) to soluble fibrin. FXIII promotes cross-linking of fibrin, resulting in the formation of blood clots. Anticoagulants inhibit thrombosis by disturbing the coagulation cascade. Warfarin inhibits the production of vitamin K–dependent coagulation factors (including FII, FVII, IX, and X) in the liver. Heparin primarily promotes the activity of AT-III. DOACs, including apixaban, edoxaban, and rivaroxaban, function as the inhibitor of FX, while dabigatran is the inhibitor of thrombin. Novel anticoagulants, including ISIS 416858 and BAY2976217, are FXI antisense oligonucleotides, while BAY1213790 is the monoclonal antibody to FXI_a_. AT-III, antithrombin III; DOAC, direct oral anticoagulant; FVII, coagulation factor VII; FXI, factor XI; FXI_a_, activated factor XI; TF, tissue factor.

As an important part of physiological hemostasis, coagulation is a complex process involving a series of cascade reactions between coagulation factors, which finally transform fibrinogen into insoluble fibrin, causing blood clotting (Figure 1).^1^ Abnormalities in coagulation factors lead to coagulation disorders, resulting in thrombosis or bleeding.^1^

As a blood filter and the most highly perfused organ, the kidney plays an important role in regulating hemodynamics and blood components, which directly affects coagulation processes.^2^ In fact, thrombosis is common in patients with nephrotic syndrome (NS) or CKD^3–5^ or patients undergoing RRTs.^6^ While patients with ESKD are threatened with bleeding.^7^ However, improper anticoagulant or procoagulant therapies may induce adverse effects, such as anticoagulation-related nephropathy (ARN), heparin-induced thrombocytopenia (HIT), or bleeding, which trouble clinicians.^8,9^ Over the decades, researchers have conducted extensive mechanism studies on the crosstalk between nephropathy and coagulopathy,^5,10^ which provide guidance for clinical treatment to some extent. However, the complex interactions between the kidney and the coagulation system are still not fully understood.

Currently, there is no review to systematically summarize and analyze the significance and shortcomings of these findings. Therefore, we review the pathogenesis and treatment of coagulation abnormalities in nephropathy, as well as the manifestations and countermeasures of adverse effects resulting from anticoagulant therapies in nephropathy patients. We emphasize the current problems in clinical practice and the corresponding deficiencies in basic research and analyze the direction of future basic research efforts on the basis of existing studies, as well as the improvement programs that could be considered in clinical treatment.

### NS

NS refers to a series of kidney diseases with similar clinical and laboratory features, which are mainly characterized by proteinuria, edema, hypercholesterolemia, and hypoalbuminemia. According to different pathological features, NS can be identified as membranous nephropathy (MN), minimal change disease, focal and segmental glomerulosclerosis, and membranoproliferative glomerulonephritis.^11^ Thromboembolism is the most serious complication of NS, which is life-threatening. The thrombosis incidence is highest in MN (approximately 37%) and lower in other pathological types of NS (with a cumulative incidence of 24%). Venous thromboembolism (VTE) is the most common type of thrombosis, and coronary thrombosis was also detected.^4,11^

#### Mechanism of Thrombosis in NS

It was reported that complicated VTE in NS was mainly caused by the disorders of coagulation and fibrinolytic proteins, while arterial thromboembolism primarily resulted from platelet hyperreactivity.^12^

**Figure 2:**
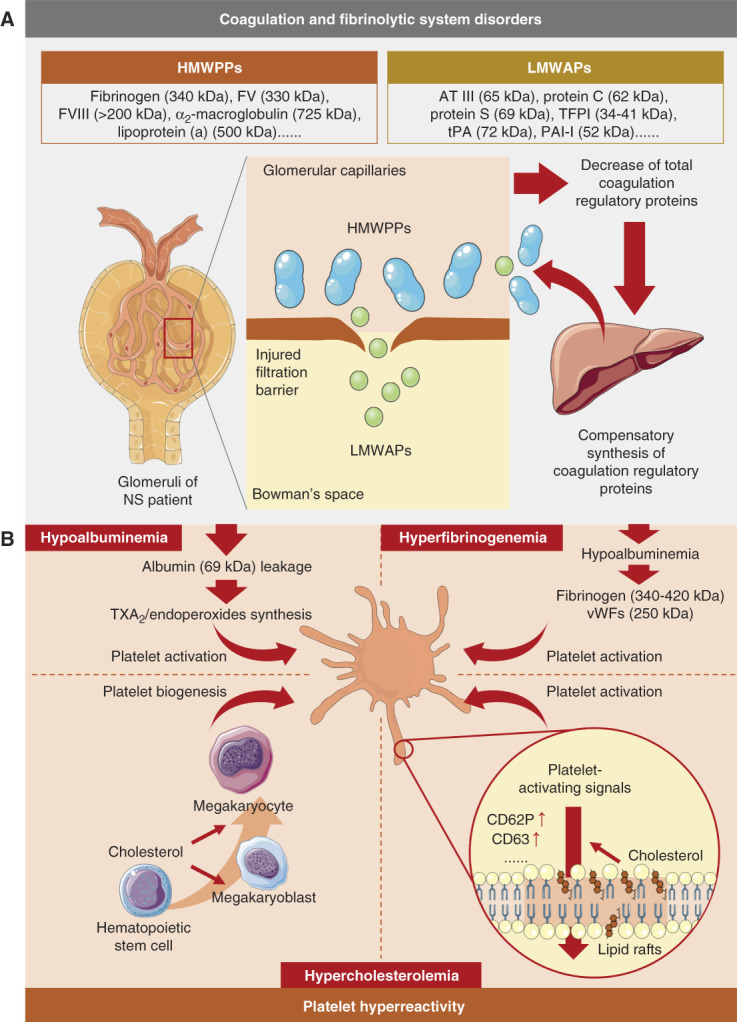
**Mechanism of thrombosis in NS.** (A) The injuries in the glomeruli filtration barrier led to the leakage of LMWAPs and retention of HMWPPs in plasma, resulting in a decrease in total coagulation regulatory protein levels. These processes induced compensatory synthesis of coagulation regulatory proteins in the liver, which further promoted the accumulation of HMWPPs in plasma, resulting in hypercoagulability. (B) Filtration barrier injuries also induced the leakage of albumin and hypoalbuminemia, which promoted TXA_2_ and endoperoxide synthesis (top left) and compensatory synthesis of fibrinogen and vWFs (top right), resulting in platelet activation. Complicated hypercholesterolemia in NS promoted platelet biogenesis by enhancing the signaling of cell surface receptors located in lipid rafts (bottom left). Hypercholesterolemia also upregulated signal transduction proteins in the lipid raft of platelets, resulting in platelet activation (bottom right). HMWPP, high molecular weight procoagulant protein; LMWAP, low molecular weight anticoagulant protein; NS, nephrotic syndrome; vWF, von Willebrand factor.

Disorders of the coagulation and fibrinolytic proteins were caused by the breakdown of the permselectivity barrier of the glomeruli capillary wall in the kidney of patients with NS. Low molecular weight anticoagulant proteins were lost from altered glomeruli (Figure 2A), whereas high molecular weight procoagulant proteins (HMWPPs) were retained in the plasma (Figure 2A).^13,14^ These processes led to a decrease in the total concentration of proteins that regulate the coagulation system, which enhanced the compensatory synthesis of both low molecular weight anticoagulant protein and HMWPP. The net effect was the accumulation of HMWPP in the blood, resulting in a hypercoagulable state that promoted thrombosis. Clinical data showed that the more severe the NS, the higher the incidence of thrombosis, which was consistent with the above mechanism.^13^

Platelet hyperreactivity in NS was mainly caused by hypoalbuminemia, hyperfibrinogenemia, and hypercholesterolemia (Figure 2B).^15,16^ Hypoalbuminemia resulted from the loss of albumin (69 kDa) from the altered glomeruli (Figure 2B, top left). Physiologically, albumin inhibited platelet aggregation by binding to arachidonic acid and preventing its metabolism into thromboxane A_2_ (TXA_2_) and endoperoxides. As a consequence, hypoalbuminemia resulted in the excessive production of TXA_2_ and endoperoxides, leading to platelet hyperreactivity.^17^ In addition, hypoalbuminemia also led to the compensatory synthesis of fibrinogen and von Willebrand factors (vWFs) in the liver, resulting in hyperfibrinogenemia that promoted platelet hyperreactivity (Figure 2B, top right).^14^ Hypercholesterolemia was one of the metabolic consequences of NS, which was confirmed as the promotor of platelet biogenesis and activation. It was reported that in various hematopoietic effector cells, membrane receptor signaling pathways located in lipid rafts were enhanced in response to membrane cholesterol accumulation. Therefore, the accumulation of cholesterol in the plasma membrane enhanced platelet biogenesis in the megakaryoblast (Figure 2B, bottom left) and procoagulant signaling transduction in the platelets (Figure 2B, bottom right), which promoted thrombosis. Indeed, numerous clinical studies detected increased platelet count and platelet hyperactivity in patients with NS, which confirmed the important role of platelets in thrombosis in patients with NS.^18–23^

#### Prophylactic and Therapeutic Anticoagulant Therapies in NS

Prophylactic and therapeutic anticoagulation is necessary for patients with NS,^24,25^ while thrombolytic therapy is only considered in patients with massive pulmonary embolism and severe bilateral deep vein thrombosis due to the high risk of bleeding.^13^

**Figure 3:**
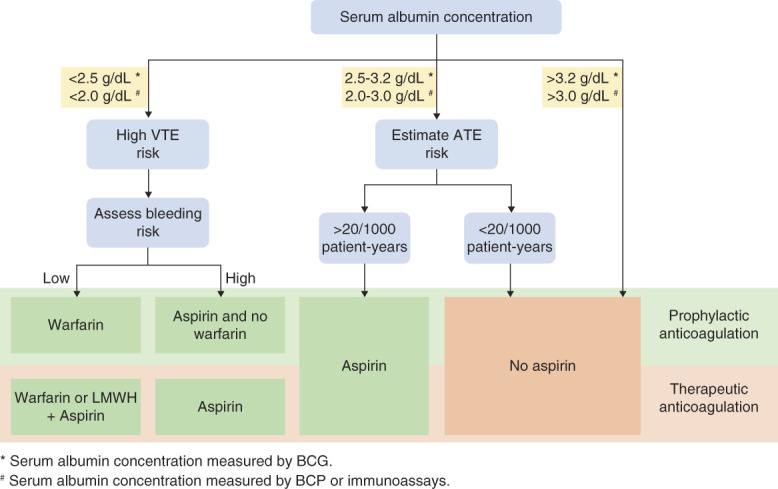
**Algorithm for prophylactic and therapeutic anticoagulation in patients with MN.** The formulation of an anticoagulant regimen is determined by serum albumin concentration. If the serum albumin concentration is <2.5 g/dl (by BCG) or <2.0 g/dl (by BCP), VTE risk should be considered; if the assessed bleeding risk is low, warfarin is considered for prophylactic anticoagulation, and warfarin or LMWH+aspirin are considered for therapeutic anticoagulation. If the serum albumin concentration is 2.5-3.2 g/dl (by BCG) or 2.0-3.0 g/dl (by BCP), ATE risk should be assessed; if the estimated ATE risk >20/1000 patient-years, aspirin is considered; otherwise, aspirin should not be used. If serum albumin concentration is >3.2 g/dl (by BCG) or >3.0 g/dl (by BCP), aspirin should not be used. KDIGO recommends an online tool (https://www.med.unc.edu/gntools/bleedrisk.html) to calculate the risk of bleeding in patients with MN. ATE, arterial thromboembolism; KDIGO, Kidney Disease Improving Global Outcomes; LMWH, low-molecular-weight heparin; MN, membranous nephropathy; VTE, venous thromboembolism; BCG, bromocresol green; BCP, bromocresol purple.

Low-molecular-weight heparin and warfarin are commonly used for prophylactic and therapeutic anticoagulation in patients with NS.^24^ But the advantages and disadvantages of these two drugs have not been compared. With the continuous accumulation of clinical data, arterial thromboembolism was also considered to need attention in patients with NS.^24,26^ Therefore, antiplatelet agents were used to prevent and treat thromboembolism in patients with NS. The Kidney Disease Improving Global Outcomes guidelines suggested aspirin may be considered for patients with MN.^27^ However, the risk of bleeding persists in patients with NS exposed to anticoagulants; thromboembolism risk and bleeding risk must be balanced during anticoagulation processes. As MN is the most common cause of NS, the Kidney Disease Improving Global Outcomes provides an algorithm for the formulation of prophylactic and therapeutic anticoagulant regimens for patients with MN (Figure 3), which might also be referenced for other pathological types of NS.^24,27^

In addition, only a few studies reported the effectiveness of direct oral anticoagulants (DOACs) in preventing thrombosis in patients with NS (Table 1). But excretion and plasma protein binding rates varied greatly among DOACs (Table 1).^33^ It is still controversial whether DOACs are suitable for patients with NS. Therefore, we do not recommend DOACs as the first choice for anticoagulation in patients with NS.

**Table 1:** Pharmacology of direct oral anticoagulants and their efficacy in patients with nephrotic syndrome

DOACs	Renal Excretion Rate	Fecal Excretion Rate	Plasma Protein Binding Rate	Efficacy in NS
Dabigatran	80% (iv)	—	35%	Preventing carotid thrombosis in patients with MN^28^
Apixaban	27%	Approximately 50%	87%	Preventing thromboembolism in patients with NS.^29^ But recurrent VTE might occur in patients with MN^30^
Edoxaban	50%	50%	55%	Inhibiting recurred renal VTE in patients with NS after warfarin treatment^31^
Rivaroxaban	66%	7%	92%–95%	Inhibiting VTE in patients with NS with low AT-III level^32^

DOACs, direct oral anticoagulants; NS, nephrotic syndrome; iv, intravenous injection; MN, membranous nephropathy; VTE, venous thromboembolism; AT-III, antithrombin III.

Faced with the current treatment dilemma, novel anticoagulants or antiplatelet drugs are urgently needed for patients with NS. Interestingly, statins were proven to not only ameliorate kidney injury but also prevent thrombosis in patients with NS.^34,35^ These efficacies might come from the inhibitory effect of stains on hypercholesterolemia, which reversed cholesterol-induced platelet hyperreactivity, as described above. Given the dual therapeutic effects of statins on NS and thrombosis, it may be an ideal choice for antiplatelet in patients with NS. More clinical studies are needed to clarify the benefits and shortcomings of statins in preventing thrombosis.

### CKD

CKD develops from a series of underlying kidney diseases. It is characterized by decreased GFR (<60 ml/[min·1.73 m^2^]) and elevated renal injury markers (such as serum creatinine and urea nitrogen). CKD significantly increases the risk of thrombosis,^5^ especially in patients complicated with atrial fibrillation (AF), which has been recognized as an important cause of stroke.^36,37^

#### Mechanism of Thrombosis in CKD

Elevated thrombosis risk in patients with CKD mainly resulted from coagulation and fibrinolytic protein disorders, endothelial dysfunction, and platelet hyperreactivity.

**Figure 4:**
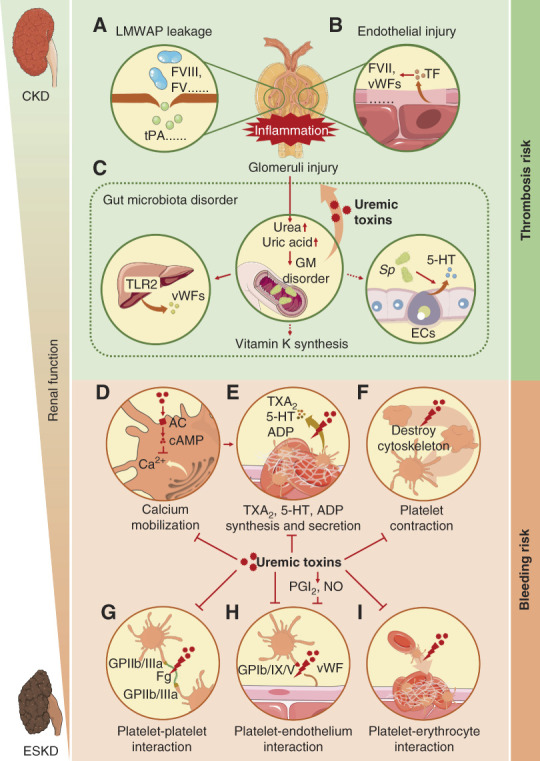
**Mechanism of thrombosis in CKD and bleeding in ESKD.** (A) The injuries in the glomeruli filtration barrier in CKD resulted in the leakage of LMWAPs and retention of HMWPPs, which promoted coagulation. (B) Glomeruli endothelial injuries in CKD led to the release of TF which triggered the extrinsic coagulation pathway. (C) Glomeruli injuries in CKD led to an accumulation of urea and uric acid in the gut, causing GM disorder, which on one hand enhanced the production of uremic toxins, resulting in glomeruli inflammation and injury; on the other hand, promoted vWFs, vitamin K, and 5-HT production. (D) Uremic toxins in ESKD activated AC and promoted cAMP production, which inhibited calcium mobilization, resulting in platelet dysfunction. (E) Uremic toxins and calcium mobilization abnormality inhibited the synthesis and secretion of TXA_2_, 5-HT, and ADP in platelets, leading to platelet dysfunction. (F) Uremic toxins destroyed the cytoskeleton of platelet, which disturbed platelet contraction, inhibiting platelet aggregation. (G) Uremic toxins inhibited platelet–platelet interaction by blocking the combination of fibrinogen (Fg) and GPIIb/IIIa, resulting in platelet aggregation dysfunction. (H) PGI_2_ and nitric oxide (NO) induced by uremic toxins and uremic toxins themselves inhibited platelet–endothelium interaction by blocking the combination of vWF and GPIb/IX/V, resulting in platelet aggregation dysfunction. (I) Uremic toxins inhibited platelet–erythrocyte interaction, resulting in platelet aggregation dysfunction. 5-HT, 5-hydroxytryptamine; AC, adenylate cyclase; GM, gut microbiota; HMWPP, high molecular weight procoagulant protein; LMWAP, low molecular weight anticoagulant protein; NO, nitric oxide; PGI_2_, prostacyclin; TF, tissue factor; TXA_2_, thromboxane A_2_; vWF, von Willebrand factor.

Coagulation and fibrinolytic protein disorders in patients with CKD were characterized by the upregulation of procoagulant proteins, especially coagulation factor VIII, vWFs, and plasminogen activator inhibitor-I.^38,39^ However, patterns of change in these proteins were not the same as that in patients with NS. Current views hold that these disorders resulted not only from glomeruli injury–induced accumulation of HMWPPs as in NS (Figure 4A) but also from the release of tissue factor (TF) and coagulation activation caused by inflammation-induced vascular endothelial injury (Figure 4B).

Platelet hyperactivity and endothelial dysfunction were caused by uremic toxins that were mainly derived from the gut microbiota (GM).^40^ It was reported that the decrease in GFR and impairment of renal tubular secretion in CKD resulted in increased production of nitrogen compounds (such as urea and uric acid) which were excreted partly through the intestine, but no urinary tract. In the gut, urea was converted to ammonium, leading to pH elevation and mucosal damage, resulting in GM disorder (Figure 4C, middle).^40^ GM disorder caused protein assimilation impairment, promoting the synthesis of uremic toxins.^40^ These uremic toxins were called thrombolome. Thrombolome can be divided into three groups: dietary tryptophan, phenylalanine/tyrosine, and choline/phosphatidylcholine (Table 2).^40^ Dietary tryptophan can combine with aryl hydrocarbon receptor (AhR) and downregulated stress-induced phosphoprotein 1 homologous and U-box containing protein 1 (STUB1). STUB1 plays an important role in TF degradation, which ubiquitinated and degraded TF in uremia environment.^42^ Dietary tryptophan-induced downregulation of STUB1 led to retarded TF degradation, which enhanced exogenous coagulation pathways.^49^ In addition, indoxyl sulfate and indole-3-acetic acid (two kinds of dietary tryptophan) could induce oxidative stress and inflammation, which also promoted platelet activation.^41,43^ Phenylalanine/tyrosine includes phenylacetylglutamine, p-cresol sulfate, and p-cresol glucuronide. Phenylacetylglutamine has been demonstrated to induce platelet hyperreactivity by activating *α*2A-, *α*2B-, and *β*2-receptors.^46^ But the mechanisms of platelet activation induced by p-cresol sulfate and p-cresol glucuronide remain unclear. It was suggested that they might promote platelet activation by promoting the release of phosphatidylserine (PS)-positive microparticles (MPs) from endothelial cells.^47^ Choline/phosphatidylcholine includes trimethylamine *N*-oxide which triggered platelet activation primarily by promoting 1,4,5-trisphosphate–mediated Ca^2+^ release.^48^

**Table 2:** Types and functions of thrombolome in CKD

Origin	Types	Functions
Dietary tryptophan	Indoxyl sulfate	a. Inhibiting endothelial cell proliferation and damaging endothelial progenitor cells by inducing oxidative stress and inflammation^41^ b. Increasing TF/FVII levels by upregulating the AhR-STUB1-TF axis^40,42^ c. Activating platelets by inducing oxidative stress-mediated p38 MAPK activation^43^
	Indole-3-acetic acid	a. Inducing COX-2 expression by activating AhR/p38 MAPK/NF-κB signaling pathways, which led to inflammation and endothelial injury^44^ b. Increasing TF/FVII levels by upregulating the AhR-STUB1-TF axis^40,42^ c. Activating platelets by inducing oxidative stress-mediated p38 MAPK activation^43^
	Kynurenine pathway–derived uremic toxins	a. Inducing inflammation and oxidative stress by activating AhR, which caused endothelial dysfunction and TF overexpression^45^ b. Causing coagulation factors disorder^40^^,^^a^
Phenylalanine/tyrosine	Phenylacetylglutamine	Inducing platelet hyperreactivity by activating *α*2A-, *α*2B-, *β*2-adrenergic receptors^46^
	P-cresol sulfate	Promoting MP release from endothelial cells^47^^,^^a^
	P-cresol glucuronide	Promoting MP release from endothelial cells^47^^,^^a^
Choline/phosphatidylcholine	Trimethylamine *N*-oxide	Promoting IP_3_-mediated Ca^2+^ release, leading to platelet hyperreactivity^48^

TF, tissue factor; FVII, coagulation factor VII; AhR, aryl hydrocarbon receptor; STUB1, stress-induced phosphoprotein 1 homologous and U-box containing protein 1; p38 MAPK, p38 mitogen-activated protein kinase; NF-κB, nuclear factor-kappaB; MPs, microparticles; IP_3_, 1,4,5-trisphosphate.

a Indicates that the underlying mechanism has not been elucidated.

In addition to uremic toxins, GM disorder could also promote hepatic vWF production by activating toll-like receptor 2 (Figure 4C, left) and might promote 5-hydroxytryptamine biosynthesis in enterochromaffin cells by upregulating tryptophan hydroxylase (Figure 4C, right).^40,50–52^ It was also reported that GM disorders may lead to increased vitamin K synthesis, but the evidence remains limited (Figure 4C, middle).^40^ These processes also contributed to thrombosis.

#### Management of Thrombosis in CKD

Patients with CKD are prone to AF (with a prevalence of 16%-21%), which greatly increases the risk of stroke.^53^ Therefore, anticoagulation is necessary for patients with CKD.^53–55^ Currently, international guidelines permit the use of warfarin and DOACs for patients with CKD.^56,57^ Dabigatran, apixaban, edoxaban, and rivaroxaban have been extensively reviewed for their safety and efficacy in patients with CKD.^57,58^ Compared with warfarin, DOACs cause a lower incidence of severe intracranial hemorrhage, eliminating the need for drug monitoring, which simplifies the treatment of patients with CKD with VTE or AF.^59^ But the differences between DOACs have not been compared. With the continuous decline of renal function, bleeding risk is elevated in patients with CKD because of the accumulation of uremic toxins. Therefore, appropriate dosage adjustments according to renal function are required for warfarin or DOACs used in patients with CKD (see detail in Table 3).^60^

**Table 3:** Dosage adjustment for anticoagulants applied in patients with CKD^53,60^

eCrCl (ml/min)	Warfarin	DOACs			
		Dabigatran	Apixaban	Edoxaban	Rivaroxaban
>95	Adjusted dose for INR 2–3	150 mg b.i.d.	5 mg b.i.d.^a^	60 mg QD^b^^,^^c^	20 mg QD
51–95	Adjusted dose for INR 2–3	150 mg b.i.d.	5 mg b.i.d.^a^	60 mg QD^c^	20 mg QD
31–50 (25–50)^d^	Adjusted dose for INR 2–3	150 mg b.i.d. or 110 mg b.i.d.^b^	5 mg b.i.d.^a^	30 mg QD^c^	15 mg QD
15–30	Adjusted dose for INR 2–3 (consider)	75 mg po b.i.d.^e^	2.5 mg b.i.d. (consider)	30 mg QD^c^ (consider)	15 mg QD (consider)
<15 (not on dialysis)	Equipoise on the basis of observational data and meta-analysis	Not recommended	2.5 mg po b.i.d.^e^	Not recommended	15 mg QD^e^
<15 (on dialysis)	Equipoise on the basis of observational data and meta-analysis	Not recommended	2.5 mg po b.i.d.^e^	Not recommended	15 mg QD^e^

eCrCl, estimated creatinine clearance; DOACs, direct oral anticoagulants; INR, international normalized ratio; b.i.d., twice daily; QD, once daily; po, per os.

a 2.5 mg twice daily if two of the three criteria are met: creatinine ≥1.5 mg/dl, body weight ≤60 kg, aged 80 years or older.

b Application of this dosage in patients with CKD has not been approved by Food and Drug Administration.

c The dose is halved if any of the three criteria are met: estimated creatinine clearance of 30 approximately 50 ml/min, body weight ≤60 kg, concomitant use of verapamil or quinidine.

d Estimated creatinine clearance values referenced for dose adjustment of apixaban.

e This application is recommended by Kidney Disease Improving Global Outcomes but lacks clinical safety or efficacy data.

Given the shorting coming of warfarin and DOACs, novel anticoagulants are urgently needed for patients with CKD. Because the AhR-STUB1-TF axis played important roles in promoting thrombosis (Table 2), blocking the AhR-STUB1-TF axis has been shown to effectively improve hypercoagulable state without increasing the risk of bleeding in patients with CKD.^49^ Targeting the AhR-STUB1-TF axis to develop anticoagulants may avoid the bleeding risk comes from DOACs, which is a direction worth making efforts in the future.

### ESKD

Patients with CKD eventually develop ESKD (GFR <15 ml/[min·1.73m^2^]) with the decline of renal function.^61^ But unlike CKD, patients with ESKD are usually threatened by bleeding.

#### Mechanism of Bleeding in ESKD

Studies indicated that uremic toxin accumulation–induced platelet dysfunction was the main cause of bleeding in patients with ESKD.^5,61,62^ As adenylyl cyclase activators, uremic toxins could upregulate cAMP that inhibited platelet calcium mobilization, which suppressed platelet activation (Figure 4D).^63^ Studies found that uremic toxins could inhibit the synthesis and release of TXA_2_, 5-hydroxytryptamine, and ADP in platelets (Figure 4E),^64,65^ which may also be one of the causes of platelet dysfunction, but the underlying mechanism has not been clarified. Besides, uremic toxins have also been shown to inhibit actin, thereby disrupting the platelet cytoskeleton, resulting in platelet contraction, movement, and secretion dysfunction (Figure 4F).^66^ In addition, uremic toxins could inhibit the binding of GPIIb/IIIa to fibrinogen without affecting the abundance of GPIIb/IIIa on the platelet membrane, resulting in decreased platelet–platelet adhesion and signaling transduction (Figure 4G).^67^ But unlike GPIIb/IIIa, the glycocalicin subunits of GPIb on the platelet membrane were degraded by uremic toxins, which disturbed the binding of vWFs with GPIb/IX/V, leading to inhibition of platelet–vessel wall adhesion. Uremic toxins also induced nitric oxide and prostacyclin production in endothelial cells, causing platelet dysfunction (Figure 4H).^67–70^ All these effects contributed to bleeding in ESKD.

In addition, patients with ESKD were often accompanied by anemia,^71^ which may also be one of the causes of bleeding because erythrocytes are important in moving platelets toward the vascular wall to promote platelet aggregation (Figure 4I).^61^ Anemia in ESKD is mainly caused by decreased secretion of erythropoietin in the kidney, shortened lifespan of erythrocytes, and inflammation.^71^ Because erythropoietin is synthesized primarily in the kidney, renal failure in ESKD results in reduced erythropoietin synthesis and subsequent dyserythrogenesis.^72,73^ Meanwhile, patients with ESKD were usually accompanied by metabolic disorders of vitamin B12 and folic acid, which further aggravated dyserythrogenesis.^74^ Shortened lifespan of erythrocytes was primarily caused by uremic toxins. Uremic toxins caused oxidative and inhibited Ca^2+^ pump in erythrocytes which led to cytoplasm Ca^2+^ overload, resulting in erythrocyte apoptosis.^73^ Inflammation inhibited erythropoiesis by inhibiting the synthesis of erythropoietin, upregulating soluble erythropoietin receptors, and inducing erythroid progenitor apoptosis.^71^ Inflammation also accelerated erythrocyte destruction by activating macrophages.^71^ In addition, inflammation exhibited inhibition effects on the expression of transferrin and disturbed the ferric metabolism in erythrocytes, which may also be the cause of anemia.^73^

#### Management of Bleeding in ESKD

The managements of bleeding in ESKD are mainly based on its pathogenesis. For platelet dysfunction, dialysis is the first option because it removes uremic toxins from the plasma. Estrogen can also be used, which inhibits the production and release of nitric oxide from vascular endothelial cells, thus enhancing platelet–vessel wall interaction.^75^ For coagulation disorders, infusion of cryoprecipitate is always applied because it is rich in coagulation factor VIII, fibrinogen, fibronectin, and vWFs.^76^ Desmopressin is also an ideal choice because it can stimulate the release of vWFs.^77^ For anemia, transfusion of packed erythrocytes or application of recombinant erythropoietin can be chosen. The benefits and drawbacks of these treatments are shown in Table 4.

**Table 4:** Benefits and adverse effects of different therapies for bleeding in patients with ESKD

Therapies	Application	Benefits	Adverse Effects
Peritoneal dialysis^61^	The preferred treatment for patients with ESKD who are at high risk of bleeding or have active bleeding	Anticoagulant therapy is not required	Peritonitis and loss of nutrients
Hemodialysis^61^	The only option for patients who are not suitable for peritoneal dialysis because of insufficient clearance or intraperitoneal surgery	Mild or no anticoagulation can reduce the risk of bleeding	Thrombosis
Applying estrogen^61^	Controlling bleeding in patients with advanced or severe kidney failure. Suitable for both male patients and female patients	Taking effect within 6 h and lasting for 2 wk by intravenous injection (0.6 mg/kg daily for 4–5 d) or taking effect within 2 d and lasting for 5 d by oral administration (25–50 mg/d). Especially suitable for patients who need continuous bleeding control Transdermal estradiol patches (50–100 *μ*g/d, twice per week) are available, whose effects last longer, which could relieve the pain of patients	Fluid retention, hypertension, and liver injury
Transfusion of cryoprecipitate^61^	Only used for the emergency treatment of acute and severe bleeding	Rapidly inhibiting bleeding in patients with uremia	Therapeutic effects are short-lived, and not all patients respond Carrying the risk of infection^76^
Applying DDAVP^61^	Controlling bleeding in patients with uremia and preventing bleeding before renal biopsy	Intravenously administered at a dosage of 0.3*µ*g/kg can inhibit bleeding within 1 h; the effect lasts for 8 h. Could also be given subcutaneously Could be given by intranasal route which is well-tolerated and safe	Repeated administration may cause rapid depletion of vWFs, leading to hypotension, tachycardia, and thrombosis in some cases
Transfusion of packed erythrocytes^78^	The main means to correct anemia before the use of erythropoiesis-stimulating agent	Bleeding time can be shortened by infusion of concentrated erythrocytes to a hematocrit of 30%^61^	Blood volume overload, hyperkalemia, iron overload, blood-borne infection, fever, and allogenic sensitization^79^
Application of recombinant erythropoietin^78^	Maintaining the hemoglobin/hematocrit levels of patients with ESKD^61^	Reducing the need for erythrocyte transfusions. Reducing fatigue and improving quality of life^79^	Increased risk of adverse cardiovascular outcomes^79^ Iron overload^79^
Applying TXA^80^	Should only be used in patients with severe, life-threatening bleeding that has failed to be controlled by other evidence-based treatments	A single small dose of TXA (7.5 mg/kg) intravenously could reduce the need for blood transfusion^81^ Could be safely used to treat severe hematuria in patients with CKD and PKD^80^ Could effectively treat massive hemorrhage of the upper digestive tract in hemodialysis patients^82^	Causing ureteral thrombosis and cortical necrosis, leading to acute renal failure. Contraindicated in patients with chronic kidney injuries^83^ Carrying the risk of systemic epilepsy because it could cross the blood–brain barrier and is neurotoxic^83^

DDAVP, desmopressin; vWFs, von Willebrand factors; TXA, tranexamic acid; PKD, polycystic kidney disease.

In addition, secondary hyperfibrinolysis resulting from the hypercoagulability of CKD is also a concern in patients with ESKD. Fibrinolytic drugs such as tranexamic acid (TXA) have been confirmed to shorten bleeding time and improve platelet function in most patients with ESKD.^84^ But TXA may accumulate in renal insufficiency and has not been shown to be more effective than commonly used therapies.^85^ Therefore, it was suggested that intravenous TXA should only be considered in acute cases where other treatments have failed to respond satisfactorily.^85^

### RRT

RRT refers to dialysis and kidney transplantation. Patients undergoing RRT are at high risk of thrombosis.^57,86^ During dialysis, the formation of blood clots may block the circulation circuit, interrupting the dialysis process, and even endangering the patient's life.^57^ While the formation of graft thrombosis in kidney transplant recipients is one of the main causes of graft loss, which has become a concern for clinicians.^86^

#### Mechanism of Thrombosis in Dialysis

Thrombosis during hemodialysis was mainly caused by excessive activation of the contact pathway of the coagulation system and platelets.^87,88^

**Figure 5:**
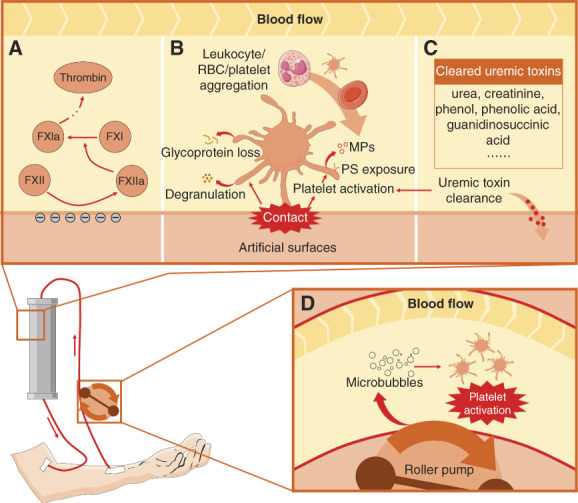
**Mechanism of thrombosis in hemodialysis.** (A) Contact between FXII and artificial surface activated the intrinsic pathway, which promoted thrombin production, resulting in enhanced coagulation. The negative charge on the artificial surface might contribute to the activation of FXII. (B) Contact between platelets and artificial surfaces induced platelet degranulation, PS exposure, PS-positive MP shedding, and glycoprotein loss. These effects eventually led to platelet activation and leukocyte, RBC, and platelet aggregation. (C) Hemodialysis cleared uremic toxins that induced platelet dysfunction, which promoted platelet activation. (D) Roller pump in the extracorporeal circuit induces the formation of microbubbles which promoted platelet activation. MP, microparticle; PS, phosphatidylserine; RBC, red blood cell.

Activation of the contact pathway during hemodialysis has been demonstrated.^87,88^ Numerous studies have found significant increases in FXIIa levels in the blood of patients undergoing hemodialysis.^87,88^ FXIIa led to a surge in the production of thrombin which promoted blood clotting.^87,88^ Activation of FXII may be related to its contact with artificial surfaces because polyvinyl chloride, polyacrylonitrile, polymethylmethacrylate, cellulose triacetate, cuprophane, and polysulfone have been shown to induce FXII activation and thrombin production.^88^ However, the mechanism of contact pathway activation has not been well elucidated. It was only known that the negative charge on the artificial surface was conducive to the activation of FXII to some extent (Figure 5A).^89^ It was reported that FXII inhibitors, such as corn trypsin inhibitor, exhibited inhibitory effects on thrombin production induced by polyvinyl chloride membrane in whole blood or plasma.^90^ Therefore, elucidating the mechanism by which artificial surface activates FXII is of great significance for the development of novel anticoagulation agents or artificial surfaces that can effectively avoid thrombosis during hemodialysis.

The changes in platelet function during hemodialysis were complex because they were regulated by artificial surfaces, uremic toxins, and hemodynamics.^57,61,91^ It was reported that the contact of platelets with the artificial surface directly caused platelet activation and degranulation (Figure 5B).^61,92^ But artificial surface also led to the loss of platelet membrane glycoproteins at the same time (Figure 5B).^92^ Therefore, in the process of hemodialysis, a temporary platelet dysfunction usually occurred before platelet activation.^92^ Meanwhile, uremic toxins that may cause platelet dysfunction were removed during hemodialysis, which reversed platelet dysfunction (Figure 5C).^61^ In addition, platelets were directly exposed to the roller pump in the extracorporeal circuit; the changes in hemodynamics might induce the formation of microbubbles that promoted platelet activation (Figure 5D).^91^ These effects resulted in platelet hyperreactivity and thrombosis.

Differently, thrombosis is rare in patients undergoing peritoneal dialysis because of the lack of artificial surfaces.^93^

#### Mechanism of Thrombosis in Kidney Transplantation

The underlying diseases and perioperative treatments may be the causes of thrombosis in kidney transplant recipients.^94^

Before kidney transplantation, underlying diseases (such as NS) and preoperative treatments (such as dialysis) always resulted in a hypercoagulable state. Patients with ESKD usually required invasive treatments or experienced arteriosclerosis that led to endothelial injury.^95^ Besides, the use of diuretics increased blood viscosity and slowed blood flow.^3^ These disturbances led to coagulation and fibrinolytic system disorders, resulting in thrombosis.^96^

During kidney transplantation, physiological factors such as shorter and thinner renal veins,^97^ younger ages, and different blood types between recipients and donors may promote graft thrombosis.^98^ Prolonged ischemia during transplantation may induce acute tubular necrosis with graft edema, leading to thrombosis.^99^ Operation-induced vascular torsion, anastomotic stenosis, and endothelial injury can also lead to thrombosis.^100^

After kidney transplantation, patients usually received immunosuppressive therapies. Many immunosuppressants, such as cyclosporine, tacrolimusand, high doses of pulsed methylprednisolone, anti-CD3 antibody, thymoglobulin, mycophenolate mofetil, and azathioprine have been confirmed to disturb coagulation and fibrinolytic systems or cause vascular dysfunction, which were also an important cause of thrombosis.^101^ The mechanisms by which these immunosuppressants promoted thrombosis are described in detail in Table 5.

**Table 5:** Immunosuppressants that induced thrombosis after kidney transplantation

Immunosuppressant	Function	Prothrombotic Effects
Cyclosporine (calcineurin inhibitors)	Inhibiting *Il-2* transcription and T-cell activation	Inducing renal vascular endothelial injury through its nephrotoxicity, which promoted thromboplastin generation, FVIII activation, TXA_2_ release, platelet activation, and suppressed thrombomodulin activity, hence downregulating the protein C anticoagulant pathway^102^ Elevating PAI and LDL levels and inducing hypofibrinolysis, which increased thrombogenicity and accelerated atherosclerosis^39,103^
Tacrolimus (calcineurin inhibitors)	Inhibiting IL-2 release and T-cell activation	Inducing vascular dysfunction by causing increases in vasoconstrictive factors (such as endothelin and thromboxane), activation of the renin-angiotensin system, and decreases in vasodilative factors (such as PGE_2_, PGI_2_, and NO)^104^
High doses of pulsed methylprednisolone (corticosteroid)	Inhibiting the phagocytosis of neutrophils and inducing T-cell apoptosis^105^	Inducing overproduction of procoagulant factors^106^
OKT3 antibody (antithymocyte/antilymphocyte globulin)	Directly targeting the TCR-CD3 complex, which is presently the most potent agent to treat acute renal allograft rejection^107^	Inducing TF expression at the surface of both monocytes and endothelial cells, which activated the extrinsic coagulation pathway^107^
Thymoglobulin (antithymocyte/antilymphocyte globulin)	Inducing lymphocyte depletion in the peripheral blood by activating complement-dependent cell lysis and inducing T-cell apoptosis	Thymoglobulin was not specific for T cells; it contained antibodies directed against platelets, which could activate platelets, inducing thrombosis^108^
Mycophenolate mofetil (antimetabolite)	Noncompetitive, reversible inhibiting inosine monophosphate dehydrogenase, which is required for purine synthesis during lymphocyte activation^109^	Although mycophenolate mofetil did not affect platelet function, it may act as a cofactor in the coagulation cascade, which promoted thrombosis by facilitating the production of Leiden-mutated FV (which cannot be inactivated by protein C)^109,110^
Azathioprine (antimetabolite)	Azathioprine is converted into six-mercaptopurine which inhibits DNA synthesis, thereby inhibiting lymphocyte proliferation^111^	May induce warfarin resistance by enhancing the synthesis of vitamin K–dependent coagulation factors^111^

FVIII, coagulation factor VIII; TXA_2_, thromboxane A_2_; PAI, plasminogen activator inhibitor; PGE_2_, prostaglandin E_2_; PGI_2_, prostacyclin; NO, nitric oxide; OKT3, anti-CD3 antibody; TCR, T-cell antigen receptor; FV, coagulation factor V; TF, tissue factor.

#### Anticoagulation in Dialysis

**Figure 6:**
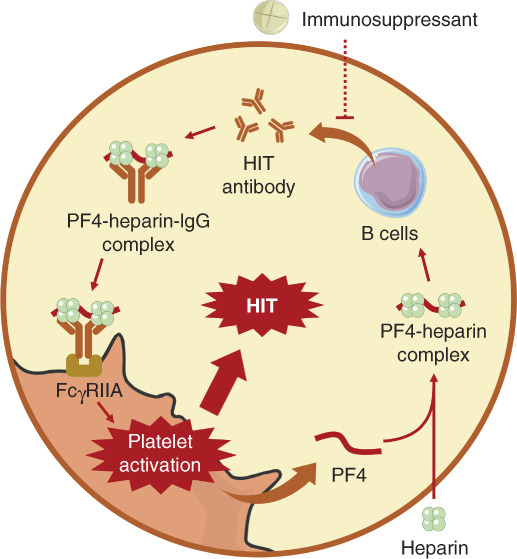
**Mechanism of HIT.** Heparin bound to PF4 to form the PF4-heparin complex that induced HIT antibody production from B cells. HIT antibodies bound to the PF4-heparin complex to form a PF4-heparin-IgG complex. PF4-heparin-IgG complex interacted with FcγRIIA, which induced platelet activation and secondary PF4 release. This circulation promoted the activation of a large number of platelets, resulting in HIT. Immunosuppressants might inhibit B cells from producing HIT antibodies, which inhibited HIT. HIT, heparin-induced thrombocytopenia; PF4, platelet factor 4.

Anticoagulation is necessary for patients undergoing continuous RRT (CRRT). At present, guidelines recommend heparin or thrombin inhibitors for anticoagulant treatment in dialysis patients (Table 6).^6,112,115^ Warfarin is not recommended for dialysis patients with AF because it may induce stroke and bleeding.^116,117^ However, the application of heparin might cause HIT (the underlying mechanism is shown in Figure 6),^9,118^ which is a severe immune response characterized by severe thrombosis with a high mortality rate. Therefore, timely HIT detection is necessary for dialysis patients receiving heparin anticoagulation. But the diagnosis of HIT is complex because it relies on clinical suspicion followed by stepwise testing, which delays the treatment of HIT.^119^ Inspiring, our team has developed the first HIT antibody assay kit in China that could be used for fast bedside HIT diagnosis, which brought benefits to patients undergoing CRRT. An effective method to avoid HIT in CRRT is to use thrombin inhibitors, but the irreversibility and narrow therapeutic window limit its application.

**Table 6:** Recommended anticoagulants for dialysis patients^6,112^

Anticoagulants	Dosages	Advantages	Disadvantages
Unfractionated heparin	5–15 IU/kg (initial) 5–10 IU/kg per hour (continuous)	Ease to use and could be reversed by protamine with a short half-life and lower cost	May induce bleeding, hypertriglyceridemia, HIT, osteoporosis, and hyperkalemia
Regional heparin with protamine	Heparin prefilter: 1000–1500 U/h Protamine postfilter: 10–12 mg/h	Lower risk of bleeding	Complexity in administration and high cost
Enoxaparin	0.15 mg/kg (initial) 0.05 mg/kg per hour (continuous)	Lower risk of HIT	Lacking reversal agent, resulting in bioaccumulation risk. Higher cost
Dabigatran	15–25 IU/kg (initial) 5 IU/kg per hour (continuous)		
Argatroban	0.1 mg/kg (initial) 0.05–0.2 mg/kg per minute (continuous)	Can be used in patients with HIT	With a narrow therapeutic window and bleeding and anaphylaxis risk.^113^ Lacking reversal agent^114^
Bivalirudin	2 mg/h		
RCA	Infused to achieve a citrate blood concentration of 3–4 mmol/L	Lower risk of bleeding and may improve inflammatory profile	Might cause metabolic acidosis because of citrate accumulation. Needing calcium monitoring

HIT, heparin-induced thrombocytopenia; RCA, regional citrate anticoagulation.

Given the defects of heparin and thrombin inhibitors, regional citrate anticoagulation (RCA) has emerged in recent years, which is reversible and more effective in anticoagulation with less bleeding and HIT risk.^120^ However, excessive RCA treatment might cause metabolic acidosis because of citrate accumulation. As a consequence, calcium monitoring is necessary for patients receiving RCA.^121^

In addition, DOACs were also used in dialysis patients, but apixaban was the only drug that has been proven to be safely used for acute VTE treatment in dialysis patients. Dabigatran and rivaroxaban are not recommended for dialysis patients because of the lack of insufficient evidence.

In recent years, the benefits of factor XI (FXI) inhibitors in dialysis patients have attracted extensive attention. Different from FX, FXI is only involved in the internal coagulation pathway (as shown in Figure 1). Because the hypercoagulable state during dialysis is mainly affected by internal pathways, targeting FXI may gain more advantages than targeting FX in the anticoagulation treatment.^122^ Several clinical trials have been conducted on the anticoagulant effects of FXI inhibitors in dialysis patients (Table 7); these inhibitors may be the dawn of dialysis patients in the future.

**Table 7:** Clinical trials on the anticoagulation effects of factor XI inhibitors in dialysis patients

Identifier	Drugs	Types	Phases	Status
NCT03358030	ISIS 416858	FXI antisense oligonucleotide	Phase 2	Completed
NCT04534114	BAY2976217	FXI antisense oligonucleotide	Phase 2	Completed
NCT04523220	BAY1213790 (osocimab)	Monoclonal antibody to FXI_a_	Phase 2	Completed

FXI, factor XI; FXI_a_, activated factor XI.

#### Anticoagulation in Kidney Transplantation

Guidelines for anticoagulant/antiplatelet treatment in kidney transplant recipients are still lacking, but continuous anticoagulation is necessary because the risk of thrombosis persists.^123^ Heparin, low-molecular-weight heparin, and warfarin have been shown to be effective in kidney transplant recipients. Interestingly, unlike dialysis patients, kidney transplant recipients undergoing heparin treatment rarely experienced HIT even if they have a history of HIT. This phenomenon might result from the inhibitory effect of immunosuppressants on the synthesis of HIT antibodies (Figure 6),^124^ which indicated that heparin may be safe for kidney transplant recipients. Antiplatelet drugs, including clopidogrel and aspirin, could also be used but is not the first choice because available evidences showed that antiplatelet drugs did not improve graft loss in kidney transplant recipients.^125^

DOACs may not be suitable for kidney transplant recipients because it is cleared by the kidney and has drug interactions with certain immunosuppressants, especially calcineurin inhibitors.^33^

### IgA Nephropathy

IgA nephropathy is an autoimmune nephropathy that is manifested by the deposition of IgA immune complexes in the mesangial region of glomeruli.^126,127^ Although the incidence of thrombosis is low, many studies have also found hypercoagulable states in patients with IgA nephropathy.^128,129^

#### Generation of Hypercoagulable States in IgA Nephropathy

**Figure 7:**
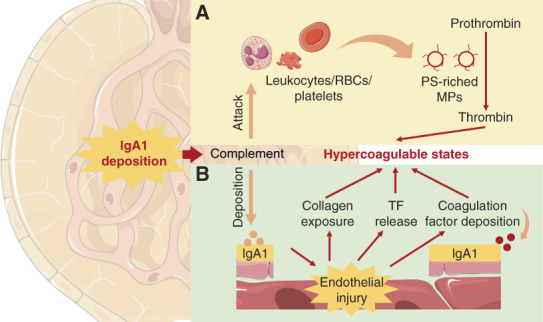
**Mechanism of the generation of hypercoagulable state in IgA nephropathy.** (A) The deposited complement attacked PS-positive MP-originating cells, leading to the shedding of PS-riched MPs, which promoted the production of thrombin, resulting in hypercoagulable states. (B) The deposited complement induced endothelial injury, which promoted collagen exposure, TF release, and coagulation factor deposition, resulting in hypercoagulable states. MP, microparticle; PS, phosphatidylserine; TF, tissue factor.

The hypercoagulable state of patients with IgA nephropathy may be associated with excessive accumulation of coagulation factors in injured glomeruli, especially coagulation factor V, coagulation factor VII, FVIII, and FIX.^130,131^ The deposition of coagulation factors may be secondary to IgA deposition. It was reported that the glomeruli capillary wall offered the first contact with circulating macromolecular IgA1, which triggered the secondary complement deposition and alternative pathway activation. Activation of complement destroyed vascular endothelium, which induced collagen exposure and TF release, resulting in coagulation activation (Figure 7B).

MPs originated from leukocytes, red blood cells, and platelets might also contribute to the hypercoagulable states in patients with IgA nephropathy. It was reported that terminal complement complex (C5b–C9) may attack MP-originating cells, pouncing PS to the outer leaflet of the plasma membrane accompanied by the shedding of MPs; MPs promoted thrombin production by providing binding sites for the assembly of the prothrombinase and tenase complexes (Figure 7A).^132^ Indeed, patients with IgA nephropathy usually showed a high level of PS-positive MPs and increased PS exposure in MP-originating cells.^133^ It was reported that the addition of MPs derived from patients with IgA nephropathy or normal ECs cultured with serum from patients with IgA nephropathy to MP-depleted plasma containing erythrocytes and leukocytes significantly reduced the median coagulation time of MP-depleted plasma, which could be restored by lactadherin (a PS blocker),^133^ confirming the procoagulant roles of MPs in patients with IgA nephropathy.

#### Benefits of Antithrombotic Agents for Patients with IgA Nephropathy

At present, there are no guidelines indicating the need for anticoagulation in patients with IgA nephropathy. But antithrombotic agents have been widely applied to patients with IgA nephropathy in China and Japan (Table 8).^150^ Several multicenter randomized controlled trials found that anticoagulants/antiplatelet drugs significantly ameliorated renal injury caused by IgA immune complex deposition (Table 8).^138,150^ These effects may be related to the downregulation of MPs by anticoagulants/antiplatelet drugs because MPs could also bind to complement C3 and induce proinflammatory cytokines in IgA nephropathy.^151^

**Table 8:** Antithrombotic agents that showed therapeutic effects on IgA nephropathy

Agents	Known Efficacy
Anticoagulation	
Danaparoid	One-week treatment with danaparoid (400 U/kg, twice daily, ip) significantly improved kidney injury in the IgA nephropathy mouse model^134^
Heparin	Continuous intravenous heparin infusion (10,000-25,000 U/d) combined with glucocorticoid (40 mg/d) improved kidney injuries by downregulating caldesmon and inhibiting mesangial cell activation in patients with IgA nephropathy^135^
Antiplatelet	
Dipyridamole	a. Dipyridamole (300 mg/d, po) significantly reduced proteinuria in patients with IgA nephropathy^139,140^
	b. Dipyridamole alone (5 mg/kg·days, po) slightly improved albuminuria in childhood IgA nephropathy^141^
Aspirin	Aspirin (100-200 mg/d) combined with eicosapentaenoic acid (1800-2700 mg/d) effectively inhibited IgA and C3 deposition in the glomeruli and improved renal function in patients with progressive IgA nephropathy^142^
Dilazep	a. Dilazep (300 mg/kg·d, oral administration at age 12-60 wk or 20-60 wk) effectively improved renal dysfunction in the IgA nephropathy mouse model^143^
	b. Dilazep significantly inhibited proteinuria and improved renal injury in patients with IgA nephropathy^144^
Ferulate	Erazine ferulate tablets combined with eucalyptol limonene pinene enteric soft capsules effectively improved kidney injury in childhood IgA nephropathy^145^
Anticoagulation+ antiplatelet	
Warfarin+ dipyridamole	Combining warfarin (a single morning dose maintains the thrombotest at 20%-50%) and dipyridamole (6 mg/kg·days, up to 300 mg/d, po) with prednisolone and mizoribine for 2 yr effectively inhibited albuminuria in severe childhood IgA nephropathy but showed a tendency to promote glomerulosclerosis^138^
Heparin+ warfarin+ dipyridamole	a. Pretreatment of continuous intravenous heparin infusion (10,000-25,000 U/d) for 8 wk followed by warfarin (1-2 mg/d) effectively enhanced the therapeutic effects of dipyridamole or ACEi/ARB combined with dipyridamole on patients with progressive IgA nephropathy^136^
	b. Combining heparin-warfarin (a single morning dose to maintain the thrombotest at 30%-50%) and dipyridamole (5 mg/kg·days, up to 400 mg/d, po) with prednisolone and azathioprine for 2 yr effectively improved glomerulosclerosis in childhood IgA nephropathy^137^
Thrombolysis	
Urokinase	a. A single injection of urokinase induced significant fibrinolytic activity, effectively improving albuminuria and/or hematuria in patients with IgA nephropathy^146^
	b. Urokinase significantly reduced deposition of C3 in patients with IgA nephropathy, but did not affect IgA and fibrin deposition^147^
	c. In patients with severe IgA nephropathy, therapeutic effects of urokinase (100,000 IU/d) combined with benazepril (10 mg/d) were superior to benazepril alone^148^
	d. Continuous urokinase therapy gained better prognoses than antiplatelet therapies in patients with moderate-to-advanced degrees of IgA nephropathy^149^

ip, intraperitoneal injection; ACEi, angiotensin-converting enzyme inhibitors; ARB, angiotensin II receptor blockers; po, per os.

However, IgA nephropathy is considered as the most common underlying disease of ARN.^152^ Warfarin,^153,154^ dabigatran,^155,156^ and aspirin^157^ have been confirmed to induce ARN in patients with IgA nephropathy. Therefore, unnecessary anticoagulation in patients with IgA nephropathy may carry the risk of aggravating kidney injuries. The use of warfarin and DOACs should be minimized if not necessary.

## ARN

ARN is defined as AKI caused by oral anticoagulants such as warfarin and DOACs.^152,158^ It is mainly manifested by impairment of the glomeruli filtration barrier with Bowman's space and tubular hemorrhage, resulting in tubular obstruction and occlusion.^159^

### Pathogenesis of ARN

The pathogenesis of ARN has not been fully elucidated. It was found that dabigatran-treated CKD rats developed ARN phenotypes, which could be enhanced by protease-activated receptor (PAR)–1 inhibitor SCH79797.^160^ These findings suggested that DOACs may be the inhibitor of PARs by inhibiting thrombin production. Because PARs provide nutritional support for endothelial cells, the glomeruli destruction hemorrhage might be caused by the inhibitory effect of DOACs on PARs.^161^ However, it was also found that application of anticoagulant therapies alone cannot cause ARN, and knockout of PAR or application of PAR-1 inhibitor vorapaxar did not induce ARN in experimental models or human patients.^161,162^ Clinical data also suggested that DOACs induced ARN only when there was significant nephron reduction, hyperperfusion, and hypertension^163^ or acute glomeruli injury.^164^ Therefore, the current views hold that ARN is the result of a second hit of anticoagulants on fragile glomeruli destroyed by the primary disease.^158^

### Treatment of ARN

**Figure 8:**
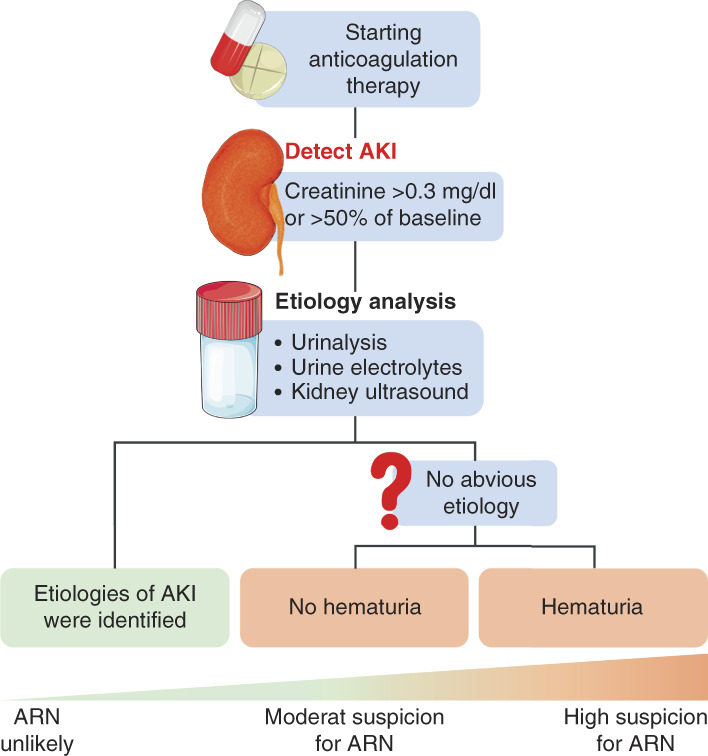
**Diagnosis of ARN.** When AKI occurs in patients undergoing anticoagulant therapy with an INR >3.0, ARN should be considered. The first diagnosis is to identify the cause of AKI by urinalysis, urine electrolytes, and kidney ultrasound. If the cause of AKI is clear, ARN could be excluded. If the cause of AKI is unclear, further diagnosis is needed to determine whether the patient develops hematuria. ARN is diagnosed if the patient develops hematuria. If the patient does not develop hematuria, the ARN is not fully determined, but the patient is always treated as ARN. ARN, anticoagulation-related nephropathy; INR, international normalized ratio.

The diagnosis of ARN is shown in Figure 8. However, treatment options for ARN are limited. One of the most effective methods is to closely monitor the patient's coagulation and renal function and adopt effective coping strategies during warfarin or DOAC treatment.^161^ For patients with ARN, treatment is supportive; control of BP and reduction of anticoagulation were commonly used.^161^ If the patient is anticoagulated with warfarin, the anticoagulation strategy should be switched to DOACs. If the patient is anticoagulated with DOACs, the dose should be reduced.^158^ In patients with primary or secondary glomerulopathy without ARN, anticoagulation should be combined with glomerular anti-inflammatory therapy to effectively prevent ARN.^158^

## Conclusion

Although the crosstalk between nephropathy and coagulation disorder has attracted the attention of clinicians and researchers for decades, practices and studies in this field are still at a relatively preliminary stage. For thromboembolism, heparin, warfarin, DOACs, and antiplatelet drugs are widely used, but adverse effects such as bleeding, HIT, and ARN have become lingering nightmares for clinicians. Owing to the lack of novel drugs, clinicians only have the option of adjusting the dosage (such as adjusting the dosage of DOACs in patients with CKD), which reduces the therapeutic effects at the same time. For bleeding in patients with ESKD, all treatment options have major defects, but there is no better alternative method at present. Encouragingly, some novel ideals point the direction for future drug development, such as inhibiting hypercholesterolemia in NS, targeting the AhR-STUB1-TF axis in CKD, and targeting FXI in dialysis. These methods bypass the limitations of classical drugs, which might bring benefits to patients with nephropathy. But more novel targets and drugs are urgently needed, which requires the joint efforts of researchers and clinicians.
